# Meaningfully reducing consumption of meat and animal products is an unsolved problem: A meta-analysis

**DOI:** 10.21203/rs.3.rs-5486065/v1

**Published:** 2025-03-19

**Authors:** Seth Ariel Green, Benny Smith, Maya B. Mathur

**Affiliations:** 1 Quantitative Sciences Unit, Department of Medicine, Stanford University.; 2 Allied Scholars for Animal Protection.

**Keywords:** meta-analysis, meat, plant-based, climate change, sustainability

## Abstract

Which interventions produce the largest and most enduring reductions in consumption of meat and animal products (MAP)? We address this question with a theoretical review and meta-analysis of randomized controlled trials that measured MAP consumption at least one day after intervention. We meta-analyze 35 papers comprising 41 studies, 112 interventions, and approximately 87,000 subjects. We find that these papers employ four major strategies to change behavior: choice architecture, persuasion, psychology, and a combination of persuasion and psychology. The pooled effect of all 112 interventions on MAP consumption is quite small (standardized mean difference [SMD] = 0.07 (95% CI: [0.02, 0.12]), indicating an unsolved problem. Interventions aiming to reduce only consumption of red and processed meat were more effective (SMD = 0.25; 95% CI: [0.11, 0.38]), but it remains unclear whether such interventions also decrease consumption of other forms of MAP. We conclude that while existing approaches do not provide a proven remedy to MAP consumption, designs and measurement strategies have generally been improving over time, and many promising interventions await rigorous evaluation.

## 1 Introduction

Global consumption of meat and animal products (MAP) is increasing [1] and is expected to continue doing so [2]. Abating this trend is vital to reducing chronic diseases caused by excessive MAP consumption and the risk of zoonotic pandemics [3–5], mitigating environmental degradation and climate change [6–8], and improving animal welfare [9, 10]. However, eating MAP is widely regarded as normal, ethical, and necessary [11, 12].

There is a vast and diverse literature investigating potential means to reduce MAP consumption. Example approaches include providing free access to meat substitutes [13], changing the price [14] or perceptions [15] of meat, and attempting to persuade people to change their diets [16]. Some interventions are associated with large impacts [17–19], and prior reviews have concluded that some frequently studied approaches, such as using persuasive messaging that appeals to animal welfare [20], may be consistently effective. A particularly high-profile strand of this literature employs choice architecture, i.e. altering the contexts in which MAP is selected [21], for instance by changing menu layouts [22, 23], placing vegetarian items more prominently in dining halls [24], or making plant-based options the default at catered meals [25]. Choice architecture could be a cheap, effective way of altering dietary behavior [26], and governments, universities, and other institutions are increasingly implementing these approaches in such settings as dining halls [27] and hospital cafeterias [28].

However, recurring design and measurement limitations compromise the literature on MAP reduction. Many interventions are either not randomized [29] or underpowered [30]. Measured outcomes are often imperfect proxies of MAP consumption, such as attitudes, intentions, and hypothetical choices [31, 32], yet behaviors often do not track with these psychological processes [33, 34] and reported preferences [35]. Additionally, many studies with comparatively large effects specifically aim to reduce consumption of red and processed meat (RPM). However, because these studies exclusively measure changes in RPM, it is unknown whether they induce substitution to other forms of MAP, such as chicken or fish [36]. Thus, treating RPM consumption as a proxy of net MAP reduction, as prior reviews have done [16, 37, 38], may cause bias. Finally, many studies measure only immediate rather than long-term effects [25, 39]. This is of special concern if subjects who are encouraged to have a single vegetarian meal later compensate by consuming more MAP, which would make an immediate outcome measurement a biased estimate of overall effects. Such compensatory effects are common in dietary studies[40–42].

In the past few years, a new wave of MAP reduction research has made commendable methodological advances in design, measurement validity, and statistical power. Historically, in some scientific fields, strong effects detected in early studies with methodological limitations were ultimately overturned by more rigorous follow-ups [43–45]. Does this phenomenon hold in the MAP reduction literature as well?

To address this question, we conducted a meta-analysis of randomized controlled trials (RCTs) that aim to reduce MAP consumption and that meet basic methodological standards [33, 46–79]. Specifically, we restricted eligibility to RCTs that measured consumption outcomes at least a single day after treatment was first administered and that had at least 25 subjects in both treatment and control (or, for cluster-assigned studies, at least ten clusters in total).

Studies in our meta-analysis pursued one of four theoretical approaches: choice architecture, psychological appeals (typically manipulations of perceived norms around eating meat), explicit persuasion (centered around animal welfare, the environment, and/or health), or a combination of psychological and persuasion messages. Interventions varied in delivery method, for example, documentary films [33], leaflets [64], university lectures [61], op-eds [58], and changes to menus in cafeterias [46] and restaurants [66, 73]. We estimated overall effect sizes as well as effect sizes associated with different theoretical approaches and delivery mechanisms. Although we find some heterogeneity across theories and mechanisms, we find consistently smaller effects on MAP consumption than previous reviews that placed fewer (if any) restrictions on studies’ outcomes and methodological rigor [20, 21, 37, 38, 80–82]. When we included studies whose methodology fell short of our inclusion criteria [11, 19, 25, 29, 39, 83–96], this considerably increased the pooled estimate. In addition, studies that only aimed to reduce RPM consumption [97–113] reported consistently stronger effects on behavior than studies aimed at reducing net MAP consumption. Overall, in contrast to previous reviews, we conclude that meaningfully reducing net MAP consumption is an unsolved problem, although many promising approaches still await rigorous evaluation.

## 2 Results

### 2.1 Results across all studies

Our meta-analysis included 35 papers comprising 41 studies and 112 separate point estimates. Each point estimate corresponded to a distinct intervention. The total sample size was approximately 87,000 subjects.

Because methodological quality is rapidly improving in this literature, the majority of eligible papers (18 of 35) were published from 2020 onwards, although the earliest was published in 2002 [71]. Among studies where treatment was assigned to individuals rather than to clusters (e.g., school classes), the median analyzed sample size per study was 132 subjects (25^th^–75^th^ percentiles: 109, 208).

We found that studies’ theoretical approaches could be grouped into four categories. **Choice architecture** studies [46, 47] (n = 2 studies with 3 estimates) manipulate aspects of physical environments to reduce MAP consumption, such as by placing the vegetarian option at eye level on a cafeteria’s billboard menu [46]. **Persuasion** studies [33, 47–69] (n = 25 studies with 77 estimates) focus on health, environmental (usually climate change), and animal welfare reasons to reduce MAP consumption. Such messages are often delivered through printed materials, such as leaflets [58, 65], booklets [51] articles and op-eds [57, 66], and videos [33, 56, 66]. Less common delivery methods included in-person dietary consultations [62], emails [50], and text messages [54]. **Psychology** studies [68, 70–74] (n = 9 studies with 12 estimates) manipulate the interpersonal, cognitive, or affective factors associated with eating MAP. The most common psychological intervention is centered on social norms seeking to alter the perceived popularity of non-MAP dishes [66, 74]. In one study, a restaurant put up signs stating that “[m]ore and more [retail store name] customers are choosing our veggie options” [73]. In another, a university cafeteria put up signs stating that “[i]n a taste test we did at the [name of cafe], 95% of people said that the veggie burger tasted good or very good!” [68]. One study told participants that people who ate meat are more likely to endorse social hierarchy and embrace human dominance over nature [71]. Other psychological interventions include response inhibition training, where subjects are trained to avoid responding impulsively to stimuli such as unhealthy food [72], and implementation intentions, where participants list potential challenges and solutions to changing their own behavior [69, 79]. Finally, some studies combine **persuasion** approaches with **psychological** appeals to reduce MAP consumption [33, 54, 60, 68, 69, 75–79] (n = 11 studies with 20 estimates). These studies typically combine a persuasive message with a norms-based appeal [68, 78] or an opportunity to pledge to reduce one’s MAP consumption [33, 79].

In our dataset, the pooled effect of all interventions is standardized mean difference (SMD) = 0.07 (95% CI: [0.02, 0.12]), *p* = .007, with some heterogeneity (standard deviation of population effects *τ* = 0.082). Given the pooled effect size and the estimated heterogeneity, we estimate that 26% of true effects are above SMD = 0.1, and just 8% are above SMD = 0.2 [114, 115].

### 2.2 Subset and moderator analyses

Stratifying by theoretical approach, pooled estimates were similar across psychology, persuasion, and persuasion and psychology (SMDs from 0.07 to 0.11; Table 1). Estimates may have been somewhat larger among the choice architecture studies (SMD = 0.21), but the sample size was much smaller (3 estimates). Within studies with a persuasion component, pooled estimates are similar for environmental appeals (SMD = 0.09, 15 studies with 28 estimates), and health appeals (SMD = 0.08, 18 studies with 30 estimates), but are smaller for appeals to animal welfare (SMD = 0.03, 16 studies with 65 estimates). We did not conduct meta-regression for theoretical approach or type of persuasion because studies with multiple interventions could occupy multiple categories, and many persuasion interventions combined multiple types of message, e.g. presenting students with both environmental and health reasons to reduce MAP consumption [61].

The 17 studies that only attempted to reduce consumption of RPM, comprising 25 point estimates, yielded a pooled effect of SMD = 0.25 (95% CI: [0.11, 0.38]), *p* = .002, *τ* = 0.201. Among these studies, we estimate that 48% of true RPM effects are above SMD = 0.2. We observe consistently small effects across categories of population (all pooled SMDs < 0.1), but more heterogeneity by region: North America, where a majority of studies took place, had an average effect of SMD = 0.04 vs. 0.14 to 0.21 for other locations. Effect sizes have broadly been declining over time, from an average of SMD = 0.16 in the 2000s to SMD = 0.05 in the 2020s.

### 2.3 Publication bias and robustness checks

**Fig. 2:**
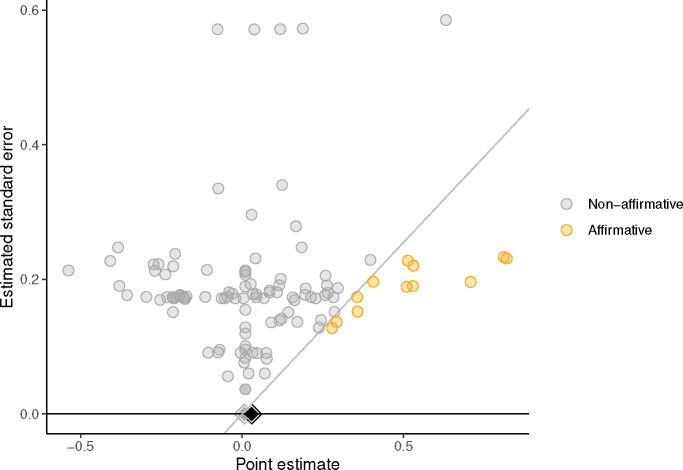
Significance funnel plot displaying studies’ point estimates versus their estimated standard errors. Orange points: affirmative studies (p < 0.05 and a positive point estimate). Grey points: nonaffirmative studies (p ≥ 0.05 or a negative point estimate). Diagonal grey line: the standard threshold of “statistical significance” for positive point estimates; studies lying on the line have exactly *p* = .05. Black diamond: main-analysis point estimate within all studies; grey diamond: worst-case point estimate within only the nonaffirmative studies.

The overall meta-analytic mean corrected for publication bias that favors significant, positive results was 0.01 (95% CI: [−0.014, 0.033]), *p* = .421 [116]; Figure 2 displays a significance funnel plot [117]. A conservative estimate that accounts for the possibility of worst-case publication bias yields an estimate of SMD = 0.02 (95% CI: [−0.01, 0.05]), *p* = .177 [117, 118] (further sensitivity checks in Supplement).

Table 2 displays subset analyses and average differences in effect size by study population, region, era of publication, and delivery method. Average differences were estimated via meta-regression.

As a robustness check, we also coded and meta-analyzed a supplementary dataset of 22 marginal studies, comprising 35 point estimates. Marginal studies were those whose methods fell short of our inclusion criteria, but typically met all but one, e.g. the control group received some aspect of treatment [11], or treatment was alternated weekly but not randomly [29] (Supplement). Expanding the meta-analytic dataset to include these marginal studies yields a pooled effect of SMD = 0.2 (95% CI: [0.09, 0.31]), *p* < 0.001. Particularly large results were found in studies that measured outcomes immediately [25] or that had smaller samples [17].

## 3 Methods

### 3.1 Study selection

Our meta-analytic sample comprises RCT evaluations of interventions intended to reduce MAP consumption that had at least 25 subjects in treatment and control (or at least 10 clusters for studies that were cluster-assigned) and that measured MAP consumption at least a single day after treatment begins. We required that studies have a pure control group receiving no treatment. We further restricted our search to studies that were publicly circulated in English by December 2023. We also made three decisions regarding study inclusion after data collection began. First, we defined a separate analytic category for studies that only targeted RPM consumption. Second, we excluded studies that did not aim to reduce either all MAP or all RPM consumption and instead sought to induce substitution from one kind of MAP to another, e.g. that encouraged treated subjects to eat fish [91]. Third, we excluded studies whose interventions left no room for participants to voluntarily decide their MAP consumption, e.g. interventions in institutions where subjects were simply served more vegetables on their plate.

Given our interdisciplinary research question and previous work indicating a large grey literature [20], we designed and carried out a customized search process. We: 1) reviewed 156 prior reviews, nine of which yielded included articles [16, 20, 21, 37, 81, 119–122]; 2) conducted backwards and forward citation search; 3) reviewed published articles by authors with papers in the meta-analysis; 4) crowdsourced potentially missing papers from leading researchers in the field; 5) searched Google Scholar for terms that had come up in studies repeatedly; 6) used an AI search tool to search for gray literature (https://undermind.ai/); and 7) checked two databases emerging from ongoing nonprofit projects that both seek to identify all papers on meat-reducing interventions. All three authors contributed to the search. Inclusion/exclusion decisions were primarily made by the first author, with all authors contributing to discussions about borderline cases.

**Fig. 3:**
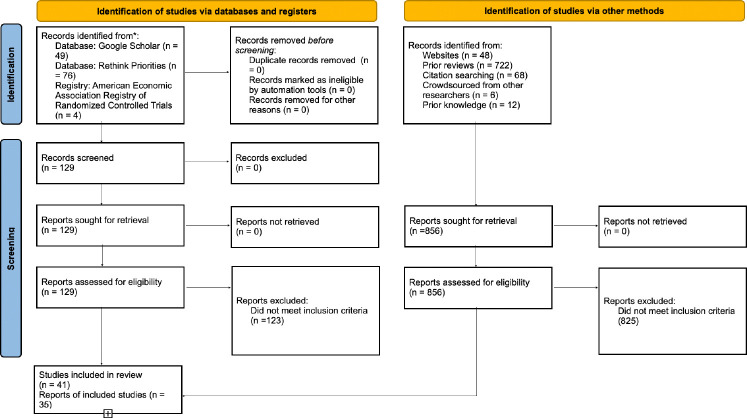
PRISMA diagram.

Figure 3 is a PRISMA diagram depicting the sources of included and excluded studies, which is detailed further in the Supplement.

### 3.2 Data extraction

The first author extracted all data. We extracted an effect size for one outcome per intervention: the measure of net MAP or RPM consumption that had the longest follow-up time after intervention. Additional variables coded included information about publication, details of the interventions, length of follow-ups, intervention theories, and additional details about interventions’ methods, contexts, and open science practices (see accompanying code and data repository for full documentation: https://doi.org/10.24433/CO.6020578.v2). When in doubt about calculating effect sizes, we consulted publicly available datasets and/or contacted authors. To assess risk of bias, we collected data on whether outcomes were self-reported or objectively measured, publication status, and presence of a pre-analysis plan and/or open data (Supplement).

All effect size conversions were conducted by the first author using methods and R code initially developed for previous papers [34, 44, 123] using standard techniques [124], with the exception of a difference in proportion estimator that treats discrete events as draws from a Bernoulli distribution (see appendix to [123] for details). As our measure of standardized mean difference, we used Glass’s Δ whenever possible, defined as $\Delta =\frac{\mu_{T}-\mu_{C}}{\sigma_{C}}$, where $\mu_{T}$ and $\mu_{C}$ respectively denote the treatment and control group means and $\sigma_{C}$ denotes the pre-treatment control group standard deviation. If the control group SD was not available, we standardized on the pooled SD. When means and SDs were not available, we converted effect sizes from: regression coefficients, eta squared, or z-scores. When there was insufficient information to calculate a specific SMD, but the text reports the result as a null, we recorded the outcome as an “unspecified null” and set it to 0.01.

### 3.3 Statistical analysis

We used <monospace>Rmarkdown</monospace> [125] and a containerized online platform [126, 127] to ensure computational reproducibility [128]. We conducted meta-analysis using robust variance estimation (RVE) methods [129] as implemented by the <monospace>robumeta</monospace> package in <monospace>R</monospace> [130, 131]. Many studies in our sample compared multiple treatment groups to a single control group. Therefore, we used the RVE method to allow for the resulting dependence between observations, as well as a standard small-sample correction.

Data analyses were largely conducted with custom functions building on <monospace>tidyverse</monospace> [132]. We assessed publication bias using selection model methods [116, 133], sensitivity analysis methods [118], and the significance funnel plot [117]. These methods assume that the publication process favors “statistically significant” (i.e., p < 0.05) and positive results over “nonsignificant” or negative results. Our sensitivity check meta-analyzes only non-affirmative results, which creates an estimate under a hypothetical “worst-case” publication bias scenario where affirmative studies are almost infinitely more likely to be published than non-affirmative studies. We conducted these analyses using functions in <monospace>metafor</monospace> [134] and <monospace>PublicationBias</monospace> [117, 118].

## 4 Discussion

Our meta-analysis of RCTs estimated a small overall effect of SMD = 0.07, along with its upper confidence bound of SMD = 0.12. Effects were also consistently small across an array of locations, study designs, and intervention categories. Some individual studies found comparatively larger effects (e.g. five studies estimated SMD > 0.5: [47, 51, 54, 62, 68]). We view these these interventions as intriguing candidates for subsequent research and replication. However, these studies’ heterogeneous theories, methods, and implementation details suggest that no singular approach, means of delivery, or message should be considered a well-validated method of reducing MAP consumption. Taken together, these findings suggest that reducing MAP consumption is an unsolved problem.

Perhaps surprisingly, our results diverged from the more positive findings of previous reviews [20, 82, 135], which are summarized in the Supplement. Our much smaller estimate likely reflects our stricter methodological inclusion criteria. For instance, of the ten largest effect sizes in a previous meta-analysis [33], nine measured attitudes and/or intentions, and the tenth came from a non-randomized design. Prior research has found that intentions often do not predict behavior [33], and reviews in other fields have found systematic differences in impacts between randomized and nonrandomized evaluations [34, 136]. Supporting this interpretation, robustness checks in which we relaxed our methodological inclusion criteria produced results similar to those of previous reviews. This possibility will need further empirical evaluation.

Another potentially surprising result is that only two choice architecture papers met our methodological inclusion criteria. Most potentially eligible papers either measured hypothetical outcomes or measured outcomes immediately after the intervention. Moreover, prior reviews that found choice architecture approaches to be consistently effective at modifying diet typically focused on foods that may have weaker cultural and social attachments than MAP, such as sugary drinks and snacks [137, 138]. We speculate that changes to how MAP is sold and consumed, by contrast, are more likely to be noticed and to engender political and cultural backlash [139].

Likewise, as our analyses show, studies aimed at reducing RPM consumption are associated with a considerably larger effect (SMD = 0.25) than those aimed at reducing all MAP consumption. Many prior reviews grouped MAP and RPM studies together, treating their outcomes as aimed at a single theoretical target [140]. However, if reductions in RPM lead to consumers’ substituting to other forms of MAP, then analyses that synthesize the two categories of outcome may produce inflated estimates of net MAP reduction. We view such substitutions as likely: many health guidelines, such as the heart-healthy diet [141], encourage reducing RPM while also encourage moderate intake of poultry and fish, both of which come with severe externalities, such as risking zoonotic outbreaks from factory farms [5] and causing land and water pollution [142]. Additionally, raising chicken and fish may lead to substantially worse outcomes for animal welfare [143]. We speculate that cutting back on RPM by substituting to other forms of MAP may be easier and more socially normative than is cutting back on all MAP. This possibility might explain the observed difference in effect sizes.

Our analyses have limitations. Relatively few studies met our methodological inclusion criteria, limiting statistical precision. Additionally, as with all meta-regression analyses, ours should not be interpreted as causal estimates of study-level moderators. That is, estimated differences in effect sizes between groups of studies do not represent the causal effects of the study characteristics (e.g., theoretical approach) on their interventions’ effects because studies’ characteristics are not randomly assigned. Finally, although our methodological inclusion criteria were more stringent than those of previous reviews, the included studies still had limitations. For example, many outcome measures in our database were coarse, such as self-reported reduction vs. non-reduction in MAP consumption as a binary variable [69]. Other studies seek to associate eating MAP with a sense of threat [77] or with endorsing social hierarchy [71] and then collect self-reported outcomes. These designs raise the possibility of social desirability bias.

Overall, this literature shows encouraging trends in methodology. First, as noted, a majority of studies in our meta-analysis have been published since 2020, indicating the field’s increasing attention to rigorous design and measurement. Second, we observe many fruitful collaborations between researchers and advocacy organizations, as shown by the large number of nonprofit white papers in our sample. Third, many promising designs and interventions still await rigorous evaluation. For instance, no study that met our criteria evaluated extended contact with farm animals [144], manipulations to the price of meat [145], activating moral and/or physical disgust [146], watching popular media such as the *Simpsons* episode “Lisa the Vegetarian” [147] or the movie *Babe* [148], and many categories of choice architecture intervention [149]. Moreover, emerging research designs help address longstanding measurement challenges, such as the possibility that interventions implemented at one time point (e.g., choice architecture at a lunch buffet) create later compensatory behavior (e.g., eating more MAP at dinner) [150]. Ultimately, our findings suggest that meaningfully reducing MAP consumption is an unsolved problem, and points to the critical importance of the field’s increasing focus on methodological rigor.

## Figures and Tables

**Fig. 1:**
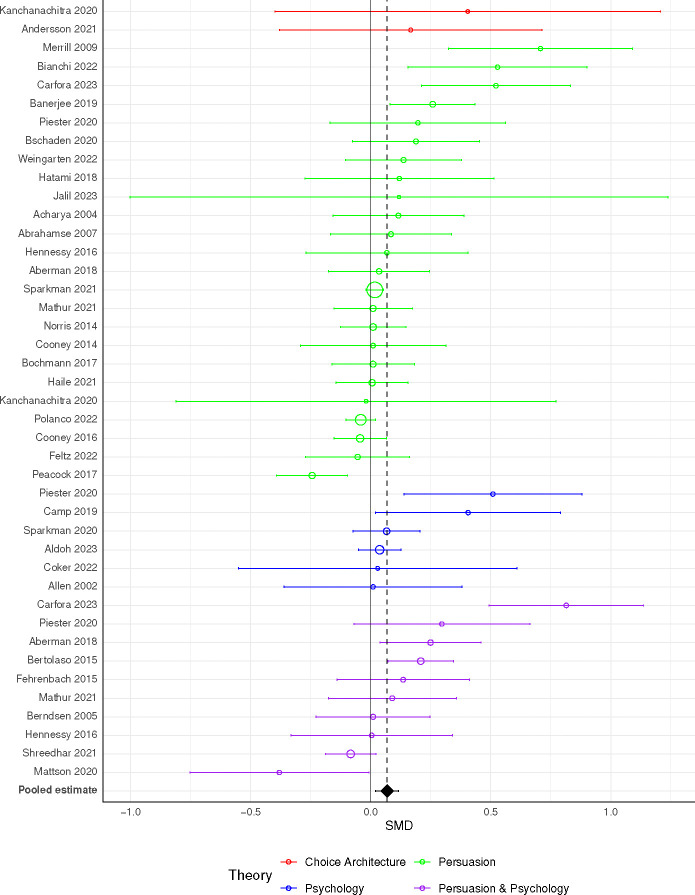
Forest plot of all meta-analyzed studies. For papers contributing multiple point estimates, the plotted point corresponds to a fixed effects meta-analysis for each paper for visual clarity. Papers employing multiple theoretical approaches are represented once per theory. Point size is inversely proportional to variance. Points are sorted within theory by estimate size. The vertical black line demarcates an effect size of zero, and the dotted line is the observed overall effect.

**Table 1:** Meta-analytic Results Overall and by Theoretical Approach

Approach	N (Studies)	N (Estimates)	SMD	95% CIs	*p* val

Overall	41	112	0.07	[0.02, 0.12]	.007
**Theory**					
Choice Architecture	2	3	0.21	[−0.99, 1.42]	.267
Psychology	19	32	0.10	[0, 0.2]	.054
Persuasion	25	77	0.07	[0.01, 0.13]	.023
Persuasion & Psychology	11	20	0.11	[−0.06, 0.28]	.189
**Type of Persuasion**					
Animal Welfare	16	65	0.03	[−0.02, 0.07]	.189
Environment	15	28	0.09	[−0.03, 0.2]	.115
Health	18	30	0.08	[−0.01, 0.17]	.068

Note that studies could occupy multiple categories for both theory and type of persuasion, that Ns for Types of Persuasion draws from both Persuasion and Persuasion and Psychology studies, and that some studies with multiple interventions are represented in multiple theoretical categories.

**Table 2:** Moderator Analysis Results

Study Characteristic	N (Studies)	N (Estimates)	SMD	95% CIs	Subset *p* value	Moderator *p* value

**Outcome**						
Meat and animal products	41	112	0.07	[0.02, 0.12]	.007	**ref**
Red and processed meat	17	25	0.25	[0.11, 0.38]	.002	.046
**Population**						
University students/staff	18	38	0.07	[−0.03, 0.16]	.139	**ref**
All ages	3	6	0.04	[−0.16, 0.25]	.361	.733
Adults	17	61	0.09	[0.01, 0.18]	.034	.714
Adolescents	3	6	0.02	[−0.4, 0.44]	.806	.686
**Region**						
North America	23	74	0.04	[−0.01, 0.08]	.142	**ref**
Europe	14	28	0.14	[0.02, 0.27]	.029	.156
Multi-region	1	4	0.21	[0.21, 0.21]	0	.000
Asia + Australia	2	5	0.13	[−0.17, 0.43]	.116	.220
**Publication Decade**						
2000s	6	8	0.16	[−0.12, 0.43]	.199	**ref**
2010s	12	31	0.07	[−0.03, 0.17]	.13	.464
2020s	23	73	0.05	[−0.01, 0.11]	.074	.369
**Method of Delivery**						
Educational materials	15	59	0.01	[−0.04, 0.07]	.566	**ref**
Online	8	22	0.16	[−0.02, 0.34]	.067	.170
Dietary consultation	2	2	0.40	[−3.36, 4.15]	.409	.441
In-cafeteria	8	13	0.10	[−0.04, 0.25]	.101	.123
Video	10	16	0.01	[−0.05, 0.07]	.485	.533

Moderation analyses by differences in outcomes, population, region, decade of publication, and delivery method. The first *p* value column tests the hypothesis that the subset of studies with a given characteristic is significantly different than an SMD of zero. The second compares effects within a given category to the reference category for that moderator.
